# Purine salvage enzymes are innate immune regulators in *Caenorhabditis briggsae* and *Caenorhabditis remanei*

**DOI:** 10.17912/micropub.biology.002371

**Published:** 2026-09-24

**Authors:** Mya G. Flannery, Mona A. Hamad, Spencer S. Gang

**Affiliations:** 1 Department of Molecular Biology, Colorado College, Colorado Springs, CO, United States; 2 School of Medicine, Emory University, Atlanta, GA, United States

## Abstract

In *Caenorhabditis elegans* , disruption of the purine salvage enzymes ADAH-1 or PNP-1 induces an innate immune program coined the Intracellular Pathogen Response (IPR). To determine if these enzymes have conserved immune regulatory roles, we utilized RNAi-competent strains of *Caenorhabditis briggsae* and *Caenorhabditis remanei* to knock down *Cbr/ Cre-adah-1* and *Cbr/ Cre-pnp-1* . In both species, knockdown of these enzymes induces expression of *Cbr/Cre-pals* genes, a hallmark of the *C. elegans* IPR. Furthermore, knockdown of *Cbr- adah-1* or *Cbr- pnp-1* promotes resistance to the microsporidia species *Nematocida parisii* . Our results suggest that perturbations to purine salvage metabolism may activate innate immune programs across *Caenorhabditis* species.

**Figure 1. Purine salvage enzymes have conserved immune regulatory roles in C. elegans , C. briggsae, and C. remanei:** **A)** Diagram of the purine salvage pathway. The enzymes ADAH-1 and PNP-1 , which negatively regulate the *C. elegans* IPR, are shown in red. ADAH-1 and PNP-1 also act on deoxy forms of nucleosides, not shown for simplicity. **B)** Representative images of *C. elegans jyIs8 [ pals-5 p::GFP; myo-2 p::mCherry] X* adults following treatment with control, *adah-1,* or *pnp-1* RNAi. *pals-5 p::* GFP is expressed when the IPR is activated; *myo-2 p::* mCherry is a co-expression marker in the pharynx. Scale bar = 100 μm **C)** RT-qPCR of *C. briggsae mfIs42 [Cel-sid-2 + Cel- myo-2 ::DsRed]* following treatment with control, *Cbr-adah-1* , or *Cbr-pnp-1* RNAi. Expression of *Cbr-adah-1, Cbr-pnp-1* , and three *Cbr-pals* genes ( *CBG06463* , *CBG06596* , and *CBG30948* ) was analyzed for fold change relative to control RNAi-treated animals. **D)** RT-qPCR of *C. remanei mfEx34 [Cel-sid-2 + Cel- myo-2 ::DsRed]* following treatment with control, *Cre-adah-1* , or *Cre-pnp-1* RNAi. Expression of *Cre-adah-1 , Cre-pnp-1 ,* and three *Cre-pals* genes ( *CRE18779, CRE09158,* and *CRE27419* ) was analyzed for fold change relative to control RNAi-treated animals. **E)** Representative images of *C. briggsae mfIs42* animals treated with control, *Cbr-pnp-1* , or *Cbr-adah-1* RNAi (top) or *C. elegans* wild-type and *pnp-1( jy121 )* mutants in the *jyIs8* background (bottom) stained with *N. parisii* -specific FISH probe (red-orange fluorescence). Solid black lines denote regions of worms quantified for *N. parisii* infection in **F** , while white dashed lines denote pharyngeal areas with co-expression markers that were omitted from infection quantification. Scale bar = 100 μm **F)** Quantification of *N. parisii* FISH fluorescence. ** *p* < 0.01, * *p* < 0.05, ns = not significant, one-way ANOVA with Tukey's multiple comparisons test. n = 3 independent infection experiments (73-81 animals analyzed per strain and condition total). Circles represent average *N. parisii* FISH fluorescence normalized to body area. Bar heights indicate mean values, and error bars represent standard deviations. **C, D)** **** *p* < 0.0001, ** *p <* 0.01, * *p* < 0.05, unpaired, one-tailed Welch's T-test. n = 4 independent experimental replicates; Circles represent the expression values for individual replicates. Bar heights indicate mean values, and error bars represent standard deviations.

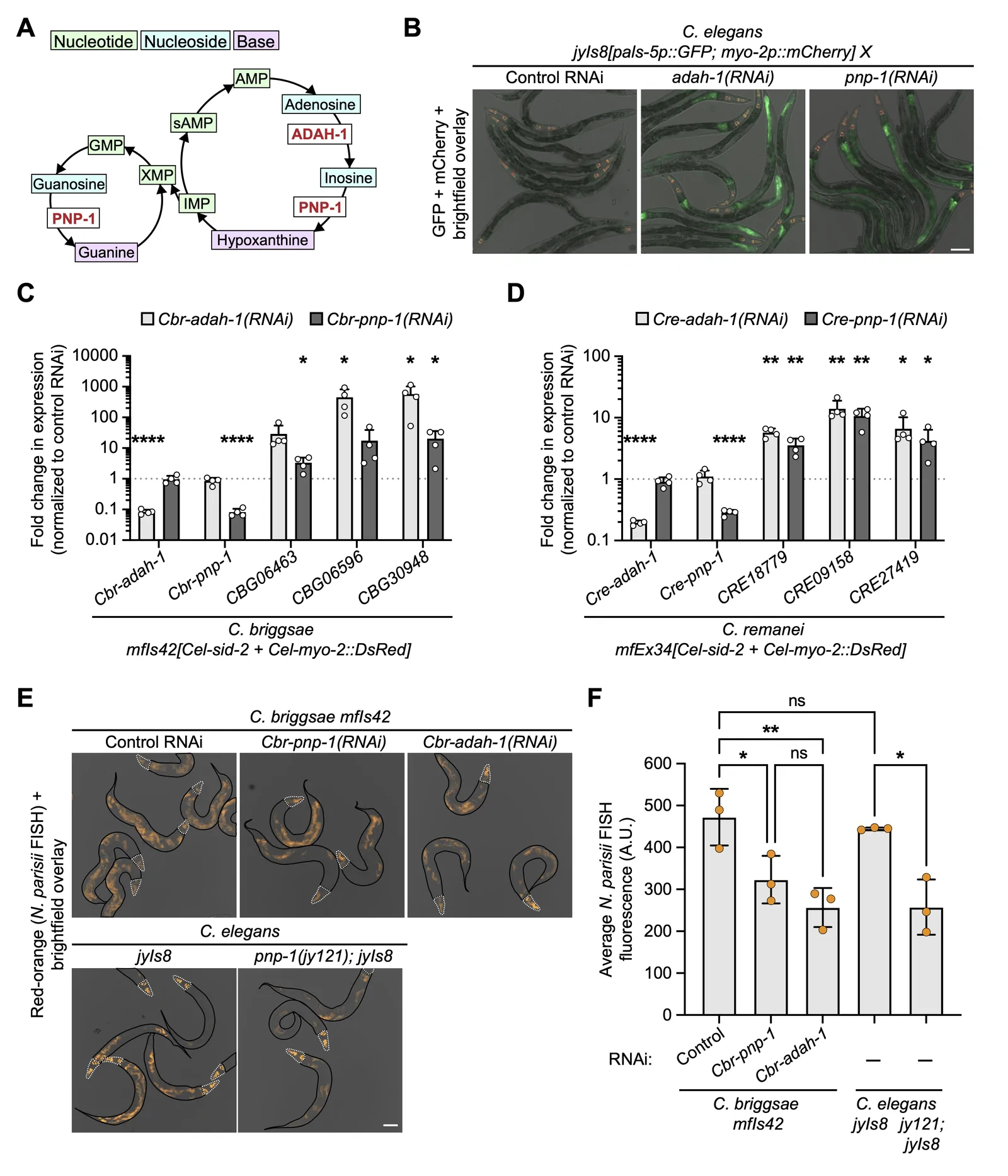



## Description

The Intracellular Pathogen Response (IPR) is an innate immune program in *Caenorhabditis elegans* involving the upregulation of a suite of genes in response to natural intracellular pathogens of the intestine, including Orsay virus and microsporidia species like *Nematocida parisii* (Sarkies et al., 2013; Bakowski et al., 2014; Reddy et al., 2019). In addition to infection, the IPR is also genetically regulated through several parallel pathways (Reddy et al., 2017; Reddy et al., 2019; Sowa et al., 2020; Lazetic et al., 2023). Two such genetic regulators are enzymes of the highly conserved purine salvage pathway, Adenosine Deaminase ( ADAH-1 , ADA in humans) and Purine Nucleoside Phosphorylase ( PNP-1 , PNP in humans), which recycle purine nucleosides and bases ( Figure 1A ) (Tecle et al., 2021; Wernet et al., 2025). Knockout or knockdown of *adah-1* or *pnp-1* induces expression of the *pals-5 p::* GFP IPR transcriptional reporter ( Figure 1B ) (Bakowski et al., 2014), as well as many other IPR genes, and promotes resistance to intracellular infections. Thus, ADAH-1 and PNP-1 act as negative regulators of the IPR (Tecle et al., 2021; Wernet et al., 2025).

While the IPR is well-defined in *C. elegans* , the extent to which this immune program is conserved or divergent among related nematode species is unclear. Infection of *Caenorhabditis briggsae* with Santeuil virus or *Nematocida* species of microsporidia induces the upregulation of several gene families in common with the *C. elegans* IPR, such as *pals* and DUF713 genes, suggesting broadly similar transcriptional responses to intracellular infection (Chen et al., 2017; Wan et al., 2022). However, whether known genetic regulators of the IPR serve similar functions outside of *C. elegans* has not been investigated.

Here, to determine whether orthologs of *adah-1* and *pnp-1* have conserved immune regulatory roles, we used *C. briggsae* and *Caenorhabditis remanei* strains transgenically modified to express *C. elegans* SID-2 , which enables uptake of dsRNA to induce systemic RNA interference (RNAi) by feeding (Winston et al., 2007; Nuez & Felix, 2012). We cloned cDNA fragments of *Cbr-adah-1* and *Cbr-pnp-1* into the L4440 RNAi plasmid vector, transformed HT115 bacteria, and performed RNAi by feeding *C. briggsae mfIs42 [Cel-sid-2 + Cel-myo-2::DsRed]* animals dsRNA targeting each gene. RT-qPCR showed knockdown of *Cbr-adah-1* and *Cbr-pnp-1* transcript levels and the upregulation of three *Cbr-pals* genes ( *CBG06463, CBG06596,* and *CBG30948* ) when compared to control RNAi-treated animals ( Figure 1C ). *CBG06596* and *CBG30948* were induced to comparable levels as previously demonstrated for *pals-5* following *adah-1* and *pnp-1* RNAi (Wernet et al., 2025). Notably, all three *Cbr-pals* genes tested were also previously shown to be upregulated during infection by Santeuil virus, *N. parisii* , or both pathogens, suggesting these genes are both genetically and infection-regulated, like *pals* genes of the *C. elegans* IPR (Chen et al., 2017; Wan et al., 2022).

Next, we cloned cDNA fragments for *Cre-adah-1* and *Cre-pnp-1* into L4440 and performed RNAi by feeding *C. remanei mfEx34 [Cel-sid-2 + Cel-myo-2::DsRed]* animals dsRNA targeting each gene. As with *C. briggsae* , we were able to knock down the expression of *Cre-adah-1* and *Cre-pnp-1* and observed upregulation of three *Cre-pals* genes ( *CRE18779, CRE09158,* and *CRE27419* ) compared with control RNAi-treated animals ( Figure 1D ). *Cre-pals* genes were not induced to the same degree as *Cbr-pals* genes (~3 to 500-fold in *C. briggsae* versus ~3-14-fold in *C. remanei* ) ( Figure 1C, D). This reduced induction could be an intrinsic property of *pals* gene family regulation in this species or the inducibility of the specific genes tested. Alternatively, we note that knockdown of *Cre-adah-1 /pnp-1* was not as effective as *Cbr- adah-1 /pnp-1* (~0.083 and 0.085-fold in *C. briggsae* versus ~0.19 and 0.28-fold in *C. remanei* ) ( Figure 1C, D). This suggests that RNAi may be less efficient in *C. remanei mfEx34 ,* leading to higher background activity of the targeted enzyme and muted upregulation of the *Cre-pals* genes. We do not attribute this effect to the extrachromosomal inheritance pattern of the *mfEx34* transgene, as this strain has previously been reported as a spontaneous integrant; we similarly observe 100% inheritance of this transgene (Nuez & Felix, 2012). Taken together, our results demonstrate that *Cbr/ Cre-adah-1* and *Cbr/ Cre-pnp-1* negatively regulate *pals* gene expression, indicating conserved immune regulatory roles for these purine salvage enzymes, like *adah-1* and *pnp-1* for the IPR.

Constitutive expression of the *C. elegans* IPR promotes resistance to intracellular infection in the intestine (Reddy et al., 2019; Tecle et al., 2021; Lazetic et al., 2023; Wernet et al., 2025). While *pals* gene expression is a useful proxy for IPR induction, only one induced *C. elegans pals* gene has been shown to confer resistance (Raman et al., 2024). Therefore, analyzing *Cbr/Cre-pals* expression after knocking down purine salvage enzymes may not fully capture whether a broader IPR-like immune program and its associated phenotypes are induced. To assess intracellular pathogen susceptibility, we performed *Cbr- adah-1 /pnp-1* RNAi and infected *C. briggsae mfIs42* animals with *N. parisii* . We focused our analysis on *C. briggsae* for two reasons: 1) more potent RNAi and induction of *pals* genes ( Figure 1C ), and 2) both *C. briggsae* and *C. elegans* are infected by *N. parisii,* which allowed for the inclusion of *C. elegans* strains as controls in our experiment; *N. parisii* cannot infect *C. remanei* (Zhang et al., 2016; Wadi et al., 2023). At 30 hours post-infection, we FISH-stained *C. briggsae mfIs42* RNAi-treated animals with a probe targeting *N. parisii* rRNA and quantified FISH fluorescence (Troemel et al., 2008). Knockdown of *Cbr-pnp-1* and *Cbr-adah-1* resulted in reduced average FISH fluorescence when compared to control RNAi-treated animals, consistent with increased microsporidia resistance ( Figure 1E, F). The degree of resistance observed in *C. briggsae* was similar to that of *C. elegans pnp-1( jy121 )* mutants when compared to wild-type animals ( Figure 1E, F) (Tecle et al., 2021). These findings further support a conserved role for purine salvage enzymes as negative regulators of an innate immune program against intracellular pathogens in a distinct *Caenorhabditis* species.

In this study, we sought to determine if purine salvage enzymes that act as negative regulators of the *C. elegans* IPR, ADAH-1 and PNP-1 ( Figure 1A, B), serve similar immune-regulatory roles in related nematode species. We cloned cDNA fragments of *Cbr/ Cre-adah-1* and *Cbr/ Cre-pnp-1* into L4440 and utilized RNAi-competent *C. briggsae* and *C. remanei* strains to knock down these enzymes. In both species, knockdown induced *pals* gene expression indicative of IPR-like immune activation ( Figure 1C, D). Additionally, knockdown of *Cbr-adah-1* and *Cbr-pnp-1* promoted *N. parisii* resistance ( Figure 1E, F). Purine salvage metabolism represents just one of several parallel genetic pathways that regulate the *C. elegans* IPR. Other regulators include members of the *pals* gene family that act in antagonistic modules (Reddy et al., 2019; Lazetic et al., 2023), *drh-1* as a sensor of dsRNA during viral infection (Sowa et al., 2020; Batachari et al., 2024), and *zip-1* as a transcription factor that acts downstream of these factors and controls a subset of IPR genes (Lazetic et al., 2022). Future work can evaluate whether these genetic regulators have broadly conserved immune regulatory roles, as assessed here for *Cbr/ Cre-adah-1* and *Cbr/ Cre-pnp-1 ,* whether these genes have divergent functions, or whether novel regulators have evolved in the diverse nematode species that are susceptible to intracellular pathogens.

## Methods

**Worm strain maintenance**

*C. elegans , C. briggsae ,* and *C. remanei* strains (Table 1) were maintained at 21 °C on Nematode Growth Media (NGM) plates seeded with *Escherichia coli* OP50-1 . RNAi experiments were performed at 22 °C on NGM plates supplemented with 1 mM Carbenicillin and 5 mM IPTG seeded with *E. coli* HT115 (DE3) carrying the desired dsRNA expression plasmids (Table 2). Seeded RNAi plates were grown in the dark at room temperature (RT) for at least 3 days before adding worms.

**IPR reporter imaging**

P0 L4s of *C. elegans* *jyIs8* were picked to 6-cm RNAi plates seeded with control, *adah-1,* or *pnp-1* RNAi. F1 adults were then picked to the same RNAi treatments. F2 adults were imaged for IPR reporter expression on a Zeiss Axioscope 7.

**Cloning**

cDNA sequences for *Cbr/ Cre-adah-1* or *Cbr/ Cre-pnp-1* were synthesized as gene fragments (Twist Biosciences) and inserted into linearized pL4440 between the T7 promoters by NEBuilder HiFi assembly (New England Biolabs). Assembled plasmids were transformed into DH5α chemically competent cells (Thermo Fisher Scientific). Candidate clones were miniprepped (Qiagen) and sequence-confirmed for the desired insertion using Sanger and PlasmidEZ whole-vector sequencing (Azenta Life Sciences). Verified plasmids were subsequently transformed into chemically competent HT115 (DE3) bacteria prepared with the Mix & Go *E. coli* Transformation Kit (Zymo Research). Genbank files for each plasmid vector generated in this study are included as Extended Data.

**RNA extraction and RT-qPCR**

For *C. briggsae* *mfIs42* , ~10-12 P0 L4s were picked to 10-cm RNAi plates seeded with control, *Cbr-adah-1,* or *Cbr-pnp-1* RNAi. ~10-12 F1 adults were then picked to 2 x 10-cm RNAi plates of the same treatments. After 3 days, F2s were enriched for L4 and adult life stages by gravity settling in M9 in a 15 mL conical tube for RNA extraction. For *C. remanei mfEx34* , a mixed-stage plate was chunked to 10-cm RNAi plates seeded with control, *Cre-adah-1 ,* or *Cre-pnp-1* RNAi. After 3 days, F1 mixed-stage worms were chunked again to 2 x 10-cm RNAi plates of the same treatments. After 3 days, F2s were enriched for L4 and adult life stages by gravity settling in M9 in a 15 mL conical tube for RNA extraction. For both species, total RNA was purified as previously described (Gang et al., 2022) and converted to cDNA using iScript (Bio-Rad). RT-qPCR was performed with iQ SYBR Green using a CFX Connect machine running CFX Maestro v1.1 (Bio-Rad). RT-qPCR data was normalized to the *Cbr/ Cre-eef-2* gene using the Pfaffl method (Pfaffl, 2001). To assess expression, *Cbr/ Cre-adah-1* and *Cbr/ Cre-pnp-1* knockdown samples were normalized to control RNAi-treated samples. RT-qPCR primers are listed in Table 3.

**Microsporidia infection**

*mfIs42* P0 and F1 generations were picked to seeded 10-cm RNAi plates as described above. ~125 F2 L4s for each treatment were then picked to 6-cm NGM plates seeded with a thin lawn of OP50-1 covering the entire surface. A mixture of 1 million *N. parisii* spores, 50 µL of 10X-concentrated OP50-1 , and M9 to a total volume of 300 µL was then top-plated, spread evenly across the surface, and dried at RT for 10 minutes. The infection plates were transferred to 25 °C for 3 h. Infected worms were washed off the plates in M9, pelleted, and transferred to 6-cm RNAi plates seeded with HT115 (DE3) to sustain knockdown of the desired genes, without additional *N. parisii* spores, and returned to 25 °C for 27 h. At 30 hpi, worms were fixed in 4% paraformaldehyde for 30 min at RT. Worms were FISH-stained with 5 ng/µL *N. parisii* -specific MicroB CF610 probe (Biosearch Technologies), imaged on a Zeiss Axioscope 7, and infection was quantified using ImageJ as previously described (Gang et al., 2022).

**Statistics**

All statistical tests were performed in GraphPad Prism version 11. The statistical test used in each experiment, number of replicates performed, and summary of test results are described in the corresponding figure legend panel. All individual data points and the complete results of statistical tests performed are included as Extended Data.

## Reagents

**Table 1. Worm Strains**

**Table:** 

**Strain**	**Genotype**	**Description**	**Source**
ERT054	*jyIs8 [pals-5p::GFP; myo-2p::mCherry] X *	Transcriptional reporter for the *C. elegans* Intracelluar Pathogen Response (IPR)	Bakowski et al. 2014
JU1018	*mfIs42 [Cel-sid-2 + Cel-myo-2::DsRed] *	*C. briggsae* AF16 sensitized for ingestion of dsRNA via expression of *C. elegans* SID-2	Nuez and Félix 2012
JU1184	*mfEx34 [Cel-sid-2 + Cel-myo-2::DSRed] *	*C. remanei* PB4641 sensitized for ingestion of dsRNA via expression of *C. elegans* SID-2	Nuez and Félix 2012
ERT789	* pnp-1( jy121 ) IV; jyIs8 [pals-5p::GFP; myo-2p::mCherry] X *	CRISPR/Cas9-mediated knockout of endogenous *pnp-1* + IPR transcriptional reporter	Tecle et al. 2021

**Table 2. Plasmids**

**Table:** 

**Plasmid**	**Genotype**	**Description**	**Source**
pL4440	pL4440 RNAi control	pL4440 RNAi feeding vector without nematode gene target inserted	Ahringer RNAi library
pL4440-pnp-1	pL4440-pnp-1	pL4440 RNAi feeding vector targeting *pnp-1*	Ahringer RNAi library, Wernet et al. 2025
pL4440-adah-1	pL4440-adah-1	pL4440 RNAi feeding vector targeting *adah-1*	Ahringer RNAi library, Wernet et al. 2025
pSSG001	pL4440- Cbr-pnp-1	pL4440 RNAi feeding vector with 647 bp of *Cbr-pnp-1* cDNA inserted targeting exons 2 and 3, isoform A. See .gb file in Extended Data for exact sequence.	This study
pSSG004	pL4440-Cbr-adah-1	pL4440 RNAi feeding vector with 700 bp of *Cbr-adah-1* cDNA inserted targeting exons 1-4, isoform A. See .gb file in Extended Data for exact sequence.	This study
pSSG015	pL4440- Cre-adah-1	pL4440 RNAi feeding vector with 450 bp of *Cre-adah-1* cDNA inserted targeting exon 5. See .gb file in Extended Data for exact sequence.	This study
pSSG016	pL4440- Cre-pnp-1	pL4440 RNAi feeding vector with 400 bp of *Cre-pnp-1* cDNA inserted targeting exon 2. See .gb file in Extended Data for exact sequence.	This study

**Table 3. qPCR Primers**

**Table:** 

**Gene Name**	**Primer Description**	**Sequence**
*Cbr-eef-2 (CBG16945) *	*Cbr-eef-2* RT-qPCR - Forward	GCTTACTTGCCTGTCAACGAGTC
*Cbr-eef-2 (CBG16945) *	*Cbr-eef-2* RT-qPCR - Reverse	CGGTGTTGGAGCGGAGA
*Cbr-adah-1 (CBG05749)*	*Cbr-adah-1* RT-qPCR - Forward	AAAGCTCAACTCAACGCTGCC
*Cbr-adah-1 (CBG05749)*	*Cbr-adah-1* RT-qPCR - Reverse	TGGCTCTCCAGCTTCAACTAGT
*Cbr-pnp-1 (CBG13501) *	*Cbr-pnp-1* RT-qPCR - Forward	TGCTAACCTGGACGCTGATCA
*Cbr-pnp-1 (CBG13501) *	*Cbr-pnp-1* RT-qPCR - Reverse	ACGAATCGAGATGCTCGCTCT
*CBG06463*	*CBG06463* RT-qPCR - Forward	ACTACCTCCGATCGGTTGAGATGT
*CBG06463*	*CBG06463* RT-qPCR - Reverse	GCAGTCAGAGGGCTCGTGTAC
*CBG06596*	*CBG06596* RT-qPCR - Forward	GCTCTCATCTGCTCTAACCGCT
*CBG06596*	*CBG06596* RT-qPCR - Reverse	ACGGTTTGGAAAAGTTGCGGAG
*CBG30948*	*CBG30948* RT-qPCR - Forward	AGCTTCCCTGAATGATGATCCA
*CBG30948*	*CBG30948* RT-qPCR - Reverse	AACGTTCCGATCAGGTCACCC
*Cre-eef-2 (CRE20767) *	*Cre-eef-2* RT-qPCR - Forward	AGGAGTCCCAAGTCACCGGAA
*Cre-eef-2 (CRE20767) *	*Cre-eef-2* RT-qPCR - Reverse	GCTTGTCCTCCGGTGTTGGAA
*Cre-adah-1 (CRE05206) *	*Cre-adah-1* RT-qPCR - Forward	GCTGCCCGTTCCTGTTTCCTA
*Cre-adah-1 (CRE05206) *	*Cre-adah-1* RT-qPCR - Reverse	TCCGGCCTGAACCAACTTCAC
*Cre-pnp-1 (CRE22695) *	*Cre-pnp-1* RT-qPCR - Forward	AAAACTGTTGGTGCCGATGCG
*Cre-pnp-1 (CRE22695) *	*Cre-pnp-1* RT-qPCR - Reverse	AGCGAGAATCCGAGCACCTTG
*CRE18779*	*CRE18779* RT-qPCR - Forward	AGCCGCTCAGCAACAAAGTCA
*CRE18779*	*CRE18779* RT-qPCR - Reverse	CCTGAGTGCAGCTCGTTCCTC
*CRE09158*	*CRE09158* RT-qPCR - Forward	GACTTTGGCGGCTAACGAGGA
*CRE09158*	*CRE09158* RT-qPCR - Reverse	AGCTTCGGCTTCACTGATAGCA
*CRE27419*	*CRE27419* RT-qPCR - Forward	CGTGTCCCACGTGCTATTGAATC
*CRE27419*	*CRE27419* RT-qPCR - Reverse	CCAATTCGTCGACGCCTTTCG

## Data Availability

Description: Genbank plasmid sequence file for pSSG001. Resource Type: Dataset. DOI: https://doi.org/10.22002/cn2g0-w7t31 Description: Genbank plasmid sequence file for pSSG004. Resource Type: Dataset. DOI: https://doi.org/10.22002/sjxqd-n0t03 Description: Genbank plasmid sequence file for pSSG015. Resource Type: Dataset. DOI: https://doi.org/10.22002/qhrk7-de075 Description: Genbank plasmid sequence file for pSSG016. Resource Type: Dataset. DOI: https://doi.org/10.22002/d9w52-jvr62 Description: Raw data and statistics summary for Figures 1C, 1D, and 1F. Resource Type: Dataset. DOI: https://doi.org/10.22002/26eec-pdn25
